# Overview of Cancer Metabolism and Signaling Transduction

**DOI:** 10.3390/ijms24010012

**Published:** 2022-12-20

**Authors:** Hee-Suk Chae, Seong-Tshool Hong

**Affiliations:** 1 Department of Obstetrics and Gynecology, Research Institute of Clinical Medicine of Jeonbuk National University, Biomedical Research Institute of Jeonbuk National University Hospital, Jeonbuk National University Medical School, Jeonju 561-712, Jeonnbuk, Republic of Korea; 2 Department of Biomedical Sciences, Jeonbuk National University Medical School, Jeonju 561-712, Jeonnbuk, Republic of Korea

**Keywords:** cancer, metabolism, aerobic glycolysis, glutamine, redox, signal transduction, anti-cancer drug, ROS

## Abstract

Despite the remarkable progress in cancer treatment up to now, we are still far from conquering the disease. The most substantial change after the malignant transformation of normal cells into cancer cells is the alteration in their metabolism. Cancer cells reprogram their metabolism to support the elevated energy demand as well as the acquisition and maintenance of their malignancy, even in nutrient-poor environments. The metabolic alterations, even under aerobic conditions, such as the upregulation of the glucose uptake and glycolysis (the Warburg effect), increase the ROS (reactive oxygen species) and glutamine dependence, which are the prominent features of cancer metabolism. Among these metabolic alterations, high glutamine dependency has attracted serious attention in the cancer research community. In addition, the oncogenic signaling pathways of the well-known important genetic mutations play important regulatory roles, either directly or indirectly, in the central carbon metabolism. The identification of the convergent metabolic phenotypes is crucial to the targeting of cancer cells. In this review, we investigate the relationship between cancer metabolism and the signal transduction pathways, and we highlight the recent developments in anti-cancer therapy that target metabolism.

## 1. Introduction

Cancer is a complex disease that is caused by somatic mutations in key genes, such as those stimulating oncogenes, inactivating tumor suppressor genes, or inactivating stability genes, which are involved in proliferative cell division [1]. The characteristics of cancer include the limitless replicative potential, avoidance of cell death, insensitivity to growth suppressors, induction of angiogenesis, and activation of invasion and metastases [2]. Researchers have conducted extensive studies on these characteristics so far; however, they have mainly conducted them from the genetic viewpoint. Recently, researchers have recognized the importance of the alteration in metabolism after the transformation into cancer. Cancer cells enable continuous growth and division by reprogramming the metabolic signal transduction pathways related to cancer metabolism to overcome environmental limitations such as limited resources and a lack of oxygen under the control of oncogenic mutations. Normal differentiated cells depend on mitochondrial oxidative phosphorylation (OXPHOS) for the energy and biomass required for cellular processes under a precisely controlled system to prevent abnormal growth, whereas cancer cells mainly depend on aerobic glycolysis, which is called “the Warburg effect” [3].

Early on, Otto Warburg argued that cancer cells do not utilize OXPHOS due to damaged mitochondria, and that they are highly dependent on aerobic glycolysis (the conversion of glucose to lactate even in oxygen-abundant environments) [4]. However, since then, there has been increasing evidence that cancer cells show flexibility in metabolism by reversibly or simultaneously using two energy metabolism methods, depending on a wide variety of tumor microenvironments: glycolysis and respiration [5,6,7]. The metabolic reprograming of cancer cells is not limited to a high rate of aerobic glycolysis. In addition to the Warburg effect, a high reliance on glutamine is another unique hallmark of cancer cells [8]. The metabolic reprograming of cancer cells is accompanied by an increased redox homeostasis, or vice versa. In this review, we aimed to enrich the overall understanding of the complexity of cancer metabolism, as well as the role of that the signaling transduction pathway plays in it. Furthermore, we intended the review to help increase the effectiveness of cancer treatment by predicting the convergent metabolic phenotypes.

## 2. Glucose Metabolism: The Warburg Effect

**Figure 1:**
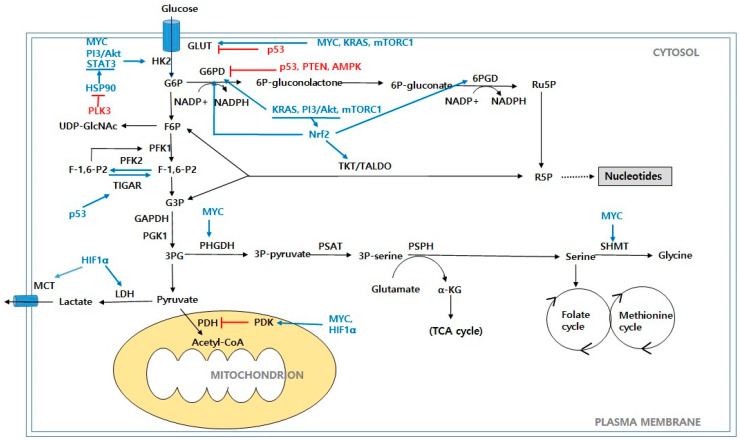
Glucose metabolism in cancer cells. Schematic representation of main metabolic pathways that contribute to production of biomass precursors, including glycolysis, PPP, HBP, and serine biosynthesis pathway. Abbreviations: GLUT: glucose transporter; HK2: hexokinase 2; G6P: glucose-6-phosphate; F6P: fructose-6-phosphate; PFK1: phosphofructokinase 1; PFK2: phosphofructokinase 2; F-1,6-P_2_: fructose-1,6-biphosphate; F-2,6-P_2_: fructose-2,6-biphosphate; TIGAR: TP53-induced glycolysis and apoptosis regulator; G3P: glyceraldehyde-3-phosphate; GAPDH: glyceraldehyde-3-phosphate dehydrogenase; PGK1: phosphoglycerate kinase 1; 3PG: 3-phosphoglycerate; PDH: pyruvate dehydrogenase; PDK: pyruvate dehydrogenase kinase; LDH: lactate dehydrogenase; MCT: monocarboxylate transporter; G6PD: glucose-6-phosphate dehydrogenase; 6P-gluconolactone: 6-phosphogluconolactone; 6P-gluconate: 6-phosphogluconate; 6PGD: 6-phosphogluconate dehydrogenase; Ru5P: ribulose-5-phosphate; R5P: ribose-5-phosphate; TKT: transketolase; TALDO: transaldolase; PHGDH: phosphoglycerate dehydrogenase; PAST: phosphoserine aminotransferase; 3P-serine: 3-phosphoserine; PSPH: phosphoserine phosphatase; α-KG: α-ketoglutarate; SHMT: serine hydroxymethyl transferase; UDP-GlcNAc: uridine diphospho-N-acetylglucosamine; PI3K/Akt: phosphoinositide 3-kinase/protein kinase B; mTORC1: mammalian target of rapamycin complex 1; Myc: c-Myc; KRAS: Kirsten Ras GTPase; HIF1α: hypoxia-inducible factors 1α; AMPK: AMP-activated protein kinase; PTEN: phosphate and tension homolog; Nrf2: nuclear factor erythroid-2-related factor 2; PLK3: polo-like kinase 3; HSP90: Heat shock protein 90; STAT3: signal transducer and activator of transcription 3.

Researchers have recently recognized an altered metabolism as one of the hallmarks of cancer [2]. Because glucose usually exists in abundance, mammalian cells mainly use it in carbon metabolism to provide energy sources and metabolites for various anabolic pathways [3]. Glucose is metabolized to the final product, pyruvate, through glycolysis. In normal resting cells, the metabolism is regulated to meet the energy needed to maintain cellular homeostasis through ATP production [9]. The glycolysis-derived pyruvate mainly enters the tri-carboxylic acid (TCA) cycle and is oxidized to produce high-efficiency ATP energy [10]. However, proliferating cancer cells must not only generate sufficient energy to support cellular replication, but they must also meet the anabolic requirements of biosynthetic macromolecules to build new cells and maintain an adequate redox balance in response to the increased production of ROS [11]. As such, cancer cells inevitably change their glucose metabolism as part of their survival and proliferation (Figure 1). Cancer cells have two biochemical characteristics: a markedly increased glucose uptake and aerobic glycolysis. Enhanced glycolysis provides metabolic intermediates and precursors for the biosynthesis of macromolecules, such as NADPH, amino acids, nucleotides, and lipids, which are required by proliferating cancer cells [12]. Furthermore, cancer cells prefer to convert most pyruvate to lactate rather than oxidize it through the TCA cycle, despite adequate oxygenation [13]. Mitochondrial OXPHOS produces 36 mol ATP/mol glucose; however, glycolysis only produces 2 mol ATP/mol glucose, which is a much smaller amount [1]. The reasons why cancer cells choose the less efficient glycolysis are as follows. First, the rate of glycolysis and the turnover of glucose into lactate is much faster than OXPHOS, which results in faster and greater ATP production [14]. Moreover, this high-rate but low-yield ATP production is an aspect of evolution, allowing cells to occupy advantageous positions when they are competing for shared energy resources [15]. High levels of glycolytic intermediates are interconnected with various metabolic pathways, particularly those that are associated with the synthesis of building blocks, and serve as substrates to support cellular anabolic reactions [16].

The metabolic reprogramming of cancer cells starts with a mutagenic emergence of oncogenes from proto-oncogenes, which induce metabolic alteration by activating the proliferative metabolic pathways and/or by mediating the metabolic gene expressions that are involved in cell proliferation. The epidermal growth factor receptor (EGFR) signaling pathway is one of the main regulators for the cell cycle that regulates growth, survival, proliferation, and differentiation in normal cells [17,18,19]. Since the EGFR signaling pathway plays a critical role in regulating the cell cycle, a mutation for constitutive activation of the genes such as EGFR, RAS family (HRAS, KRAS, and NRAS), Myc, etc., in the EGFR signaling pathway is the main driver for malignant transformation [20,21,22]. Among these oncogenes, KRAS is the best-recognized oncogene with the highest mutation rate in all cancers including highly fatal cancers such as pancreatic ductal adenocarcinoma, non-small-cell lung cancer, and colorectal cancer [23]. The oncogenic mutations of KRAS result in mitochondrial dysfunction by disruption of complex I of the electron transport system (ETS) [24]. Despite a reduced oxygen consumption as a result of mitochondrial dysfunction, the KRAS mutations increase glucose transport into cancer cells by the over-expression of GLUT1 and hexokinase 2 [25,26,27]. The inevitable consequence of elevated ATP generation after high glucose influx under mitochondrial dysfunction would be aerobic glycolysis. In fact, decreased OXPHOS, elevated glycolysis, and increased generation of reactive oxygen species have been observed as the main features in the cancer cells with oncogenic mutations of KRAS [24,28,29].

Other than genetic mutations in the oncogenes, the signaling pathway is constitutively activated in most cancers through subsequent genetic modification [21,22]. The oncogenes of the EGFR signaling pathway generally activate the cancer metabolism including anaerobic glycolysis as a result of the cell cycle activation in which Myc exerts the final role [30]. Myc, which is the final effector of the EGFR signaling pathway, promotes glycolysis by inducing the expression of glucose transporter1 (GLUT1) and lactate dehydrogenase A (LDHA) and by upregulating the glycolytic enzymes, including hexokinase 2 (HK2), phosphofructokinase (PFK), and pyruvate dehydrogenase kinase 1 (PDK1) [31,32]. Moreover, most cancers are exposed to hypoxia [33]. In hypoxia, hypoxia-inducible factor 1α (HIF-1α) dimerizes with HIF-1β and accumulates in the nucleus. The HIF-1 dimer, which is the complex of HIF-1α and HIF-1β, then binds to the hypoxia response elements (HREs) and activates the regulatory genes involved in glycolysis [34,35]. These genes include the glucose transporter GLUT1, glycolytic enzyme HK2, phosphofructokinase-1 (PFK1), fructose-bisphosphate aldolase A (ALDOA), phosphoglycerate kinase 1 (PGK1), pyruvate, pyruvate kinase (PK), and the enzymes related to lactate production, extrusion LDHA, and monocarboxylate transporter 4 (MCT-4) [36,37]. HIF-1 inactivates pyruvate dehydrogenase (PDH) via the upregulation of PDH kinases (PDKs), which inhibit the transformation of pyruvate into acetyl-CoA [38]. Researchers have observed crosstalk between Myc and HIF, and this interplay promotes cancer cell survival and growth [39]. A key tumor suppressor, p53, also plays an important role in the regulation of cellular metabolism [40]. p53 regulates the glucose absorption by repressing GLUT1 and GLUT4, as well as GLUT3 through the inhibition of nuclear factor kB (NF-ĸB) signaling [41]. p53 suppresses the MCT1 expression to inhibit the transportation of lactate [42]. In addition, p53 inhibits glycolysis through the transcriptional activation of Parkin, which degrades HIF-1α, or by downregulating HK2 [43,44]. The phosphoinositide 3-kinase (PI3K)/Akt pathway, which is one of the most frequently dysregulated pathways in cancer, is activated in response to growth factors, insulin, and cytokines and it controls key metabolic processes [45]. PI3K/Akt promotes glucose uptake and glycolysis by increasing the glucose transporter expressions such as those of HK and PFK1 [46]. HIF-1α regulates glycolysis via the PI3K/Akt/mTOR signaling pathway [47]. Tumor suppressors, such as phosphate and tension homolog (PTEN) and p53, suppress the metabolic adaptation through the negative activation of the PI3K/Akt and MAPK/ERK pathways [48,49]. Recent studies provide evidence for associations with polo-like kinases (PLKs) in regulating cancer progression, apoptosis, and metabolic processes [50,51]. The PLKs, which belong to a family of highly conserved serine/threonine kinases, comprise all five members (PLK1-5) [50]. PLKs and signal transducer and activator of transcription 3 (STAT3) exhibit cross-regulatory interactions in human cancers. PLK1 promotes the migration of lung cancer cells via STAT3 signaling [52]. Furthermore, PLK3 binds to the heat shock protein 90 (HSP90) and facilitates its degradation, which leads to a decrease of phosphorylated STATS. The STAT3 dephosphorylation further inhibits the transcriptional activation of HK2, resulting in a lower glycolytic rate in colorectal cancer [53].

The pentose phosphate pathway (PPP), which is also called the hexose monophosphate shunt, is a major glucose catabolic pathway that regulates cancer cell survival and growth by supplying ribose-5-phosphate (R5P) [54]. Furthermore, the PPP plays an important role in the cellular redox balance as a major source of NADPH [55]. Glucose is phosphorylated by HK to form glucose-6-phosphate (G6P), which is further metabolized by the PPP. The PPP has two biochemical branches: oxidative and non-oxidative. In the oxidative phase, G6P is irreversibly converted into ribulose-5-phosphate (Ru5P) by a series of G6PD and 6-phosphogluconate dehydrogenase (6PGD). In addition, NADPH produced during the oxidative phase plays a role in reductive biosynthesis and the maintenance of the redox state of the cell [56]. Ru5P yielded by 6PGD enters the non-oxidative branch and is converted to R5P and erythrose 4-phosphate, which are required for the nucleic acid synthesis and are sugar phosphate precursors for amino acid biosynthesis, respectively [57]. The non-oxidative branch recruits glycolytic intermediates, including fructose-6-phosphate (F6P) and glyceraldehyde-3-phosphate (G3P) by a series of reversible reactions, and it enables reciprocal coordination of the nutrient uptake and stress response in cancer cells [58]. Transketolase (TKL) and transaldolase (TALDO) are key enzymes that are responsible for a series of reversible reactions in the non-oxidative branch of the PPP [55]. The central role of glucose-6-phosphate dehydrogenase (G6PD),which is the first and rate-limiting enzyme in the PPP, is the generation of ribose and NADPH, which are vital for the biosynthesis of the building blocks and the maintenance of the antioxidant defenses [59]. G6PD acts as a “gatekeeper” to determine the glucose flux, acting as a partition between the glycolysis and PPP [60]. Increased NADP+ levels during oxidative stress increases the PPP flux by upregulating G6PD for the generation of NADPH and the antioxidant defense [1]. The pro-oncogenic signaling pathways that are related to the PPP are usually accelerated through G6PD [55]. Because p53 directly represses the glucose transporter genes GLUT1 and GLUT4, the loss of p53 increases both glycolysis and the PPP [61,62]. p53 induces the expression of TIGAR (TP53-induced glycolysis and apoptosis regulator) repressing the glycolysis by lowering the level of fructose-2,6-bisphosphate (F-2,6-P_2_). F-2,6-P_2_ is an allosteric activator of PFK1, and its reduction inhibits the PFK1 activity and results in a decreased glycolytic flux [63]. While p53, the p53 target gene PTEN, and AMP-activated protein kinase (AMPK) negatively regulate the PPP by suppressing the G6PD activity, the oncogenic pathways, such as KRAS, PI3/Akt, and mTORC1, increase the flux through the PPP by the upregulation of G6PD [60]. The oncogene *KRAS*^G120^ stimulates glycolysis and substantially increases the nonoxidative PPP activity in pancreatic tumors [64]. mTORC1 increases both the flux through glycolysis and the oxidative PPP through the activation of SREBP1 (a sterol regulatory element-binding protein), which is involved in the induction of the G6PD’s expression [65]. Nrf2 (nuclear factor erythroid 2-related factor 2), through its binding to antioxidant response elements (AREs) under oxidative stress or increased ROS levels, activates both the oxidative and nonoxidative PPPs by upregulating the expressions of the PPP enzymes (G6PD, 6PGD, TKT, and TALDO) [66].

After the glucose that is taken up by the cell is converted to fructose-6-phosphate, most of the intermediates are metabolized through glycolysis, and from approximately 2 to 3% are shunted to the hexosamine biosynthetic pathway (HBP), which is a metabolic branch pathway of glycolysis [67]. The nucleotide–sugar uridine diphosphate N-acetylglucosamine (UDP-GlcNAc), which is an end product of the HBP, serves as the sugar donor for *O*-linked-β-N-acetylglucosamine (*O*-GlcNAc) [68]. GlcNAcylation, which is similar to phosphorylation, is the post-translational cycling of a single *O*-GlcNAc on the hydroxyl groups of serine and/or threonine residues of the target proteins [69]. *O*-GlcNAc is added to or removed from the target proteins by two enzymes: *O*-GlcNAc transferase (OGT) and *O*-GlcNAcase (OGA), respectively [70]. *O*-GlcNAcylation elevation is implicated in a variety of cancers, including breast cancer [71], prostate cancer [72], lung cancer [73], colorectal cancer [74], and chronic lymphatic leukemia [75]. The loss of p53 increases the HBP flux and leads to the enhancement of *O*-GlcNAcylation [76]. Reducing the *O*-GlcNAcylation in prostate cancer leads to a decrease in the level of c-Myc protein *O*-GlcNAcylation and decreased c-Myc stability, which researchers have associated with decreased cancer cell growth [77]. In breast cancer, the OGT and *O*-GlcNAcylation levels are upregulated via the PI3K/mTOR/Myc pathway for tumorigenesis [78].

Researchers have observed glucose-derived metabolic fluxes into the serine biosynthesis pathway in cancer cells [79]. The glycolytic intermediate, 3-phosphoglycerate (3PG) can be diverted to produce serine, which is a major donor of carbon to the folate pool [80]. Phosphoglycerate dehydrogenase (PHGDH) is the rate-limiting enzyme that converts 3PG to serine [81]. During this serine synthesis process, phosphoserine aminotransferase (PAST) converts glutamate to α-KG, which serves as refuels for the TCA cycle; thus, the serine synthesis pathway supports the energy metabolism and anabolic processes [82]. Serine donates a one-carbon unit to tetrahydrofolate (THF) by serine hydroxymethyltransferase (SHMT1/2), which is then used for de novo purine synthesis or thymidylate synthesis [83]. Serine also transfers a one-carbon unit to homocysteine, which forms methionine [84]. Methionine is the precursor of cysteine, which is essential for the formation of glutathione, which plays an important role in redox buffering [85].

Researchers have observed the flux into this serine biosynthetic pathway through the amplification or upregulation of the PHGDH expression in many cancers [86,87]. The PHGDH expression levels in specific subtypes of cancer, such as triple-negative breast cancer [67] or estrogen-receptor-negative cancers [87], are upregulated. c-Myc is involved in the regulation of serine biosynthesis and the PHGDH expression is transcriptionally upregulated by activating c-Myc [88]. c-Myc also upregulates SHMT2 under hypoxia, and both the SHMT2 and PHGDH enzymes are positively correlated with each other in cancers such as breast cancer and neuroblastoma [89]. p53 allows cancer cells to overcome cellular stress, such as serine starvation, which preserves the cellular antioxidant capacity [90]. Glycine metabolism is also critical for tumorigenesis [91,92]. Glycine decarboxylase (GLDC) is commonly upregulated in cancers, and the mTORC1 activity regulates the posttranslational modifications of GLDC, which contribute to the modulation of glycine metabolism and tumorigenesis [93].

## 3. Tryptophan and Glutamine Metabolism

Amino acid metabolism is also significantly altered in cancer cells [94]. Although most amino acids are metabolized differently in cancer cells, the metabolisms of tryptophan and glutamine deviate most significantly in cancer cells [95]. Cancer cells unusually upregulate the expression of indoleamine 2,3-dioxygenase 1 (IDO1) that catalyzes the oxidation of L-tryptophan to N-formyl-kynurenine [96]. Overexpressed IDO1 in cancer cells makes cancer cells catabolize tryptophan leading to depletion of tryptophan. Since immune cells heavily rely on tryptophan for their immunological reaction, depletion of tryptophan by cancer cells causes the activation of apoptosis in the neighboring immune cells to cancer cells [97,98]. At the same time, the concomitant tryptophan metabolites by IDO1 such as kynurenine in cancer cells further induce T-lymphocytes to undergo apoptosis [97,98], as well as promote activation of immunosuppressive regulatory T-cells [98,99]. Although cancer cells prevent the immunological attacks in hosts by removing tryptophan, cancer cells in the same microenvironment are not affected by tryptophan depletion resulting from the accelerated tryptophan catabolism [95,96,97]. The mechanism for how cancer cells overcome IDO1-mediated tryptophan deprivation is not elucidated and is a current interesting scientific speculation.

**Figure 2:**
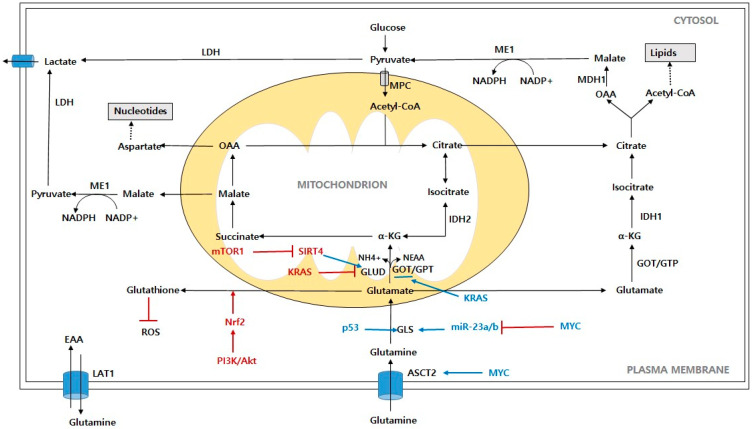
Schematic representation of glutamine contribution to cancer cells metabolism. Abbreviations: ASCT2: alanine-serine-cysteine-transporter-2; LAT1: L-type amino acid transporter; GLS: glutaminase; GLUD: glutamate dehydrogenase; α-KG: α-ketoglutarate; OAA: oxaloacetate; IDH1: isocitrate dehydrogenase 1; IDH2: isocitrate dehydrogenase 2; ME1: malic enzyme; LDH: lactate dehydrogenase; ROS: reactive oxygen species; MPC: mitochondrial pyruvate carrier; MDH1: malate dehydrogenase; NEAA: nonessential amino acid; EAA: essential amino acid; NH4+: free ammonia; PI3K/Akt: phosphoinositide 3-kinase/protein kinase B; mTORC1: mammalian target of rapamycin complex 1; Myc: c-Myc; KRAS: Kirsten Ras GTPase; Nrf2: nuclear factor erythroid-2-related factor 2; SIRT4: Sirtuin 4.

Highly proliferating cells, such as cancer cells, have an explosively increased demand for amino acids, which are an important class of major nutrients, to support their growth rate. Thus, cancer cells try to meet these demands by increasing the glutamine metabolism (Figure 2). Glutamine is the most abundant amino acid, accounting for over 20% of the free amino acids in plasma and for 40% in muscle [100]. Most tissues synthesize glutamine, which is a nonessential amino acid (NEAA); however, in periods of rapid growth or stress, the cell’s glutamine requirements increase beyond their ability to rapidly synthesize macromolecules, which makes glutamine essential [101]. Many cancer cells are addicted to glutamine, and most of them show oncogene-dependent glutamine addictions in culture [102,103]. Glutamine is emerging as an important nutrient for cancer cell proliferation and survival through the supply of carbon and nitrogen or the production of macromolecules, energy generation, and cellular homeostasis [104]. The amide group (γ-nitrogen) from glutamine contributes to both purine and pyrimidine nucleotide biosynthesis in proliferating cells [105]. Moreover, glutamine’s α-nitrogen through transaminases is transferred into various pools of nonessential amino acids (NEAAs), promoting the generation of alanine, asparagine, and serine [106]. In addition to its role as a nitrogen donor, glutamine provides a source of carbon to fuel the TCA cycle for bioenergetics and the biosynthetic requirements of cancer cells [106]. Proliferating cancer cells that display aerobic glycolysis convert glucose to lactate, which shunts glucose carbon away from the TCA cycle and fatty acid (FA) synthesis [107]. Eventually, glutamine serves as the carbon donor to maintain the TCA cycle in proliferating cells by replenishing the intermediates that act as precursors in the biosynthetic and NAPDH-producing pathways [16]. Glutamine is imported into cells through the SLC1A5 (also called ASCT2) glutamine transporter [108], and it is deaminated to glutamate by glutaminases (GLS1/2) and transported into the mitochondria [109]. Glutamate is converted into α-ketoglutarate (α-KG) in the mitochondria through oxidative deamination by glutamate dehydrogenase (GLUD) to fuel the TCA cycle [110]. Glutamine-derived aspartate is transported to the cytosol, where it is converted into oxaloacetate (OAA) by transaminase, which is subsequently converted to malate and pyruvate. Alternatively, glutamate is converted to α-KG by transaminase generated NEAAs in the cytosol or mitochondria [104]. Glutamate is also a precursor of glutathione, which serves as a redox buffer against oxidative stress, and its formation is highly dependent on glutamine [101]. Under normoxic conditions and with an adequate glucose supply, proliferating cancer cells shunt α-KG into OXPHOS to produce energy, or they shunt it into the TCA cycle to produce citrate through the condensation with glutamine-derived OAA, which is produced through a series of oxidation processes, and with glucose-derived acetyl-CoA [111,112]. Citrate is transported to the cytosol and contributes to de novo lipid synthesis. The cytosolic OAA is converted into malate through NADP-dependent malate dehydrogenase (MDH1), and the malate is converted into pyruvate by malic enzyme 1 (ME1), producing CO_2_ and NADPH [105]. TCA cycle-derived OAA can be transferred to aspartate to contribute a carbon source for nucleotide biosynthesis [16]. Glutamine anaplerosis in the TCA cycle plays an important role in the provision of the critical precursors that are used to fuel the nucleotides, NEAAs, and lipids. Under conditions of hypoxia or glucose deprivation, α-KG produces citrate and supports lipid synthesis through a reductive carboxylation by NADP-dependent isocitrate dehydrogenase 2 (IDH2) [113,114]. In addition, α-KG exported to the cytosol is reductively carboxylated into citrate via NADPH-dependent isoforms of isocitrate dehydrogenase 1 (IDH1), which in turn helps maintain lipogenesis [115]. In this way, the NADPH-producing pathways from glutamine metabolism provide the reducing power for lipid synthesis as well as the redox balance.

Oncogenes reprogram glutamine metabolism to support cancer cell growth as in the case of glucose metabolism. Since cancer cells are heavily addicted to glutamine, it is natural to observe that oncogenes, especially *KRAS*, elevate the genes associated with glutamine catabolism such *GLS*, *GLUD*, *SLC1A5*, transaminase, etc., through Myc, which is the downstream molecule of the oncogenes [116,117,118]. Myc uses glutamine as a nitrogen source to synthesize purines and pyrimidines, and it directly regulates three (CTP synthetase, PRPP amidotranferase, and carbamoyl phosphate synthetase II) of the five enzymatic steps [119]. Myc also activates the expressions of the genes involved in glutamine uptake and glutaminolysis. Myc transcriptionally binds to the promoter regions of the high affinity glutamine transporters, SLC1A5 and SLC38A5, which facilitates the glutamine uptake [120]. The bidirectional transport of glutamine through SCL1A5 and SLC7A5 is required for the activation of mTORC1 signaling [121]. Furthermore, Myc indirectly stimulates GLS through the suppression of miR-23a and miR-23b, which results in the upregulation of the glutamine metabolism [122]. Myc leads to the diversion of glucose away from the mitochondria, which is its conversion to lactate [120]. As a result, Myc enhances the cellular dependence on glutamine. Glutamine depletion induces Myc-mediated cell death via the Bax and caspase activities, which means that glutamine is a carbon source for the maintenance of mitochondrial anaplerosis [107,123]. In addition, apoptosis that is caused by glutamine depletion is rescued by oxaloacetate or pyruvate, suggesting that glutamine depletion causes apoptosis by affecting the TCA cycle [104]. The oncogene *KRAS* also plays an important role in coordinating the shift in glutamine metabolism to support tumor growth and survival by increasing the GOT1 expression and decreasing the GLUD1 expression [111]. PI3K/Akt can activate Nrf2, in which case the activation of Nrf2 upregulates the expression of glutathione synthetase and glutamate–cysteine ligase to produce glutathione [124,125]. mTOR1 promotes glutamine anaplerosis by activating GLUD by repressing silent information regulator two 4 (SIRT4), which is the mitochondrial-localized sirtuin that inhibits GLUD [126]. p53-inducible GLS2 facilitates glutamine metabolism and lowers the intracellular ROS levels to protect against oxidative stress [127].

## 4. Lipid Metabolism

**Figure 3:**
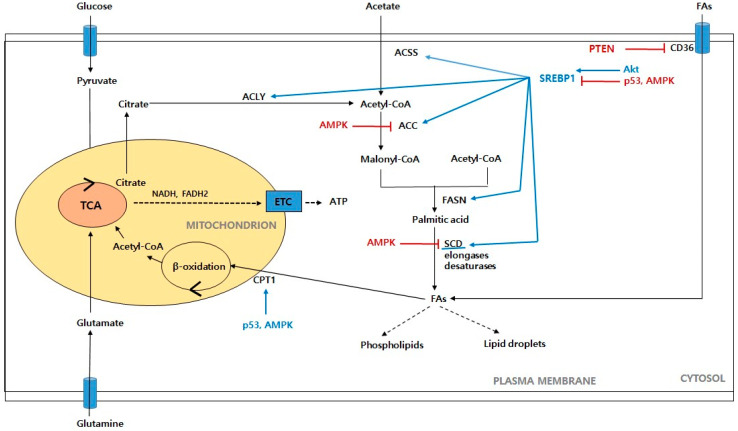
Schematic representation of fatty acid metabolism in cancer cells. Glucose-derived pyruvate enters the mitochondria to form citrate, which then moves to the cytoplasm and contributes to the de novo synthesis of fatty acids. Acetate is also another source of lipogenesis. When cellular fatty acids are in excess, the fatty acids absorbed through CD36 are converted into lipid droplets and are made available when needed. Acetate also provides reducing equivalents, such as NADH and FADH2 through β-oxidation. Finally, ATP is generated from the ETC. Abbreviations: ACSS: acetyl-CoA synthetase; ACLY: ATP citrate lyase; ACC: acetyl-CoA carboxylase; FASN: fatty acid synthase; SCD: stearoyl-CoA desaturase; FAs: fatty acids; CPT1: carnitine palmitoyl transferase 1; ETC: electron transport chain. Akt: protein kinase B; AMPK: AMP-activated protein kinase; PTEN: phosphate and tension homolog; SREBP1: sterol regulatory element-binding proteins.

Lipids are the essential components of membranes, and they are the building blocks that constitute cells [128]. Lipid metabolism dysregulation is one of the major metabolic hallmarks of cancer [129]. Cancer cells optimize their requirements through lipidomic remodeling, which includes overall alterations in the FA transport, de novo synthesis, storage as lipid droplets, and β-oxidation to produce ATP (Figure 3).

Fatty acids (FAs) are the building blocks of most lipids, and they are composed of a hydrocarbon chain with one terminal carboxyl group of varying carbon lengths and degrees of desaturation [130]. Consequently, FAs serve to regulate various biochemical processes, such as membrane biosynthesis, energy storage, and signaling pathways, in which they act as secondary messengers [131]. Tumor cells synthesize up to 95% of FAs de novo despite a sufficient dietary lipid supply, which suggests that the activity of the FA synthesis pathway is required for carcinogenesis [132,133]. A number of researchers have shown high levels of fatty acid synthesis in various human cancers and their precursor lesions, including prostate cancer [134], ovarian cancer [135], breast cancer [136], endometrium cancer [137], and colon cancer [138]. Thus, we focus on the FA metabolism in cancer cells and the oncogenic signaling pathways that support tumorigenesis and cancer progression.

Mammalian cells can obtain FAs through exogenous uptake or de novo synthesis [139]. The cellular uptake at the plasma membrane is the first step in FA utilization, and it is highly regulated through specific membrane transporters. These transporters include CD36 FA translocase, the FA transport proteins (FATPs; also known as SLC27), and plasma membrane FA-binding proteins (FABPs), all of which show increased gene and protein expression in tumors [140]. In particular, a high CD36 expression is associated with poor prognosis in cancers, such as breast, ovarian, gastric, and prostate cancers [141,142]. In PTEN-deficient prostate cancer, CD36 promotes an increased FA uptake and accelerated cancer progression, which suggests that it renders cancer cells dependent on the exogenous lipid uptake [143]. Cancer cells use the extracellular FA uptake to fill the lipid pool that is required for their rapid growth and proliferation under conditions of metabolic stress, such as hypoxia. Tumors under hypoxia exhibit an increased FA uptake through the upregulation of the FABP3/7 expression that is induced by HIF-1α, which accompanies repressed de novo FA synthesis [144]. In ovarian cancer, FABP4 is related to the supply of FAs from the surrounding adipocytes, and it is also a key determinant of the metastatic potential [145,146]. Hypoxia can downregulate miR-409-3p, thus removing its inhibitory effects on FABP4, which results in increased FABP4 levels. Therefore, the high expression of FABP4 can regulate various metabolites such as FA oxidation (FAO) and lysophospholipids and can lead to metastases in ovarian cancer.

Lipogenesis in most normal cells is usually confined to hepatocytes and adipocytes and is activated when necessary, whereas cancer cells often exhibit a shift toward de novo lipid synthesis, even in the presence of exogenous lipid sources [147]. FAs are synthesized from cytoplasmic acetyl-CoA, which is generated by ATP citrate lyase (ACLY), and acetyl-CoA synthetase (ACSS) from citrate and acetate, respectively [148]. In addition, the carbon sources from glucose and glutamine contribute to citrate production [128]. Pyruvate is converted to mitochondrial acetyl-CoA by PDH (pyruvate dehydrogenase), which is followed by citrate synthase and subsequent citrate generation [149]. As mentioned above, glutamine is converted to α-KG by GLS1 and GLS2, which is followed by IDH1 and IDH2, respectively, and subsequent citrate production [150]. Several steps are needed to convert carbons from citrate to FAs. Citrate is converted to cytoplasmic acetyl-CoA by ACLY, and then acetyl-CoA carboxylase (ACC), which is the rate-limiting enzyme of de novo lipogenesis, catalyzes the carboxylation of acetyl-CoA to malonyl-CoA [151]. Fatty acid synthase (FASN), which is a key step in the FA synthetic pathway, catalyzes the successive condensation of seven malonyl-CoA molecules and one acetyl-CoA molecule, producing 16-carbon palmitate [152]. Palmitate can be elongated and desaturated using FA elongases and stearoyl-CoA desaturases (SCD), generating additional FAs for the production of more complex lipids [130,153]. Lipogenesis is transcriptionally regulated by sterol regulatory element-binding proteins (SREBPs), which are membrane-bound transcription factors that consist of three isoforms: SREBP1a, SREBP1c, and SREBP2 [154]. SREBPs that are synthesized as inactive precursors bind to the SREBP cleavage-activating protein (SCAP) in the endoplasmic reticulum (ER) [155]. Insig-1 and Insig-2 are ER proteins that bind to the SCAP in a sterol-regulated fashion and prevent the proteolytic processing of SREBPs [155]. Under low-sterol-concentration conditions, the SCAP/SREBP complex is transported to the Golgi. In the Golgi, site-1 protease (S1P) and site-2 protease (S2P) subsequently cleave the SREBPs to release the active N terminus, which enters the nucleus and induces the transcription of the genes that are involved in lipogenesis [152,156]. As such, SREBP1 enhances lipogenesis by increasing the transcriptions of its target genes: ACLY, ACC, FASN, and SCD1. The activation of SREBPs is stimulated by the PI3K/Akt/mTOR pathway, which is the most frequently activated oncogenic signaling pathway in cancers [157]. The ACLY activity is regulated by the PI3K/Akt pathway, and Akt can directly activate ACLY [158,159]. The acetylation of ACLY increases the ACLY stability by blocking its ubiquitinylation and degradation, which leads to the promotion of de novo lipid synthesis and tumor growth [160]. Conversely, sirtuin 2 (SIRT2) deacetylates ACLY [140]. In addition, the increased phosphorylation of ACLY by Akt can enable sustained acetyl-CoA production, and thereby histone acetylation, even if nutrients are limited [161]. The activation of AMPK, which suppresses FA synthesis by phosphorylating ACC, also inhibits tumorigenesis [162,163]. The FASN activity is upregulated by the increase in the epidermal growth factor signaling via the mitogen-activated protein kinase (MAPK) and the PI3 kinase signaling cascades [164]. In addition, SCD1 contributes to the maintenance of the lipid metabolism in cancer cells, which favors cell proliferation and survival by the stimulation of the ACC activity through the inactivation of the AMPK pathway and the activation of Akt signals [165]. AMPK phosphorylates SREBPs to retain them in the ER, eventually inhibiting their transcriptional activities and suppressing lipogenesis in liver cancer cells [166]. Akt activation upregulates SREBP1 gene expression, and it also inhibits the ubiquitin-proteasome pathway degradation of SREBP1 through protein arginine methyltransferase 5 (PRMT5), inducing SREBP1 hyperactivity, which results in de novo lipogenesis and tumor growth [167]. Furthermore, mTORC2 positively regulates the lipid synthesis via the stabilization of SREBP1 by suppressing the GSK3/FBXW7-mediated degradation in cancer cells [153].

Cells convert excess lipids into triglycerides (TGs) and cholesterol esters in the ER, and they store them as lipid droplets (LDs) for use as energy generation and membrane synthesis in times of starvation [168]. Cancer cells exhibit the presence of abundant LDs, which suggests that the storage of lipids may be a common feature of malignancies [169]. TG serves as the main form of energy storage and diglyceride acyltransferase 1/2 (DGAT1/2) synthesizes TG and acyl-CoA [170]. Mitochondrial fatty acid oxidation (FAO, also known as β-oxidation) plays an important role in maintaining energy homeostasis by generating energy in the form of ATP [171]. The FAO pathway converts long-chain FAs into acetyl-CoA, which in turn generates NADPH and FADH2 through the TCA cycle, which enter the electron transport chain to produce ATP [152]. Cancers exhibit the overexpression of FAO pathway proteins [172,173]. Carnitine palmitoyl transferase 1 (CPT1), which is the rate-limiting enzyme in FAO, catalyzes the acylation of long-chain FAs and their entry into the mitochondria for FAO [174]. One of the isoforms, CPT1C, is upregulated in human lung cancer, depending on a mechanism that involves activated AMPK, and it increases the FAO and ATP generation [175]. Peroxisome proliferator activated receptor α (PPARα) is a major transcriptional regulator of FAO and extended PPARα activation causes hepatocellular carcinoma by involving the perturbation of the cell cycle and the production of ROS [152,176]. AMPK enables cancer cells to survive under metabolic stress by maintaining NADPH as well as the abovementioned function of ATP homeostasis, and this metabolic adaptation is achieved through the liver kinase B1 (LKB1)-AMPK pathway [177]. The phosphorylation and inactivation of the acetyl-CoA carboxylases ACC1 and ACC2 by AMPK maintains the NADPH levels by inhibiting FA synthesis and activating FAO, respectively [177,178]. Emerging evidence supports an additional role for p53 in lipid metabolism. The CPT1C expression is activated through the AMPK/p53 signaling pathway under metabolic stress, which leads to tumor proliferation and survival [179]. Lpin1 is essential for fat metabolism, and the ROS-mediated p53 induction of Lpin1 regulates the FAO in response to nutritional stress [180].

## 5. Redox Metabolism

ROS are the ionized oxygens and derived unstable chemicals from ionized oxygens. ROS contain unstable bonds or unpaired valence electrons that make these molecules highly reactive. Hydroxyl radical (HO·), hydrogen peroxide (H_2_O_2_), and superoxide (O_2_^−^) are typical examples of the ROS found in mammalian cells. ROS are generally highly oxidative and are therefore innately toxic in normal cells. However, ROS do not only act as toxic molecules in normal cells, but they also function as signaling molecules so that the many biological processes and functions of normal cells rely on the ROS of the redox metabolism [181,182,183]. ROS play important roles in the modulation of cell survival, death, differentiation, proliferation, growth, and migration, cytoskeletal regulation, and the cell signaling, fertilization, and production of cytokines [184,185,186]. The roles of ROS are not limited to the modulation of these cellular functions, as ROS are directly involved in various biochemical pathways. ROS react with biomacromolecules, which play important roles in inflammatory diseases, neurodegenerative diseases, the aging process, etc., to oxidize them [183,187]. In blood, ROS react with nitric oxide (NO) to produce cytotoxic reactive nitrogen species such as NO_2_ and NO_3_, which cause oxidative injury to the endothelium, which causes cardiovascular diseases [188]. The iodination of the thyroid hormone also relies on hydrogen peroxide (H_2_O_2_) as an oxidative agent [189]. Because ROS play a dual role in oxidative damages and as either signaling molecules or enzyme substrates, normal cells tightly control the cellular redox balance to maintain the optimum ROS levels [187].

**Figure 4:**
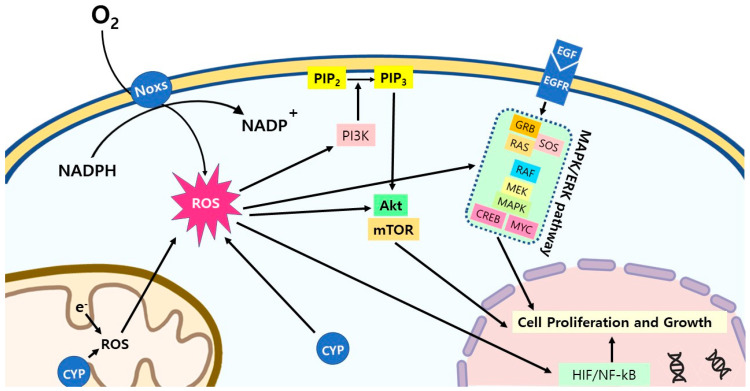
ROS signaling pathways in cancer cells. ROS activate multiple signaling pathways in cancer cells for the stimulation of the cell proliferation and growth. Abbreviations: CYP: cytochrome P450; PI3K: phosphoinositide 3-kinases; PIP_2_: phosphatidylinositol 4,5-bisphosphate; PIP_3_: phosphatidylinositol (3,4,5)-trisphosphate; AKT: protein kinase B; mTOR: mammalian target of rapamycin; EGF/EGFR: epidermal growth factor/epidermal growth factor receptor; GRB: growth factor receptor-bound protein; SOS: Son of Sevenless; RAS: Ras GTPase; RAF: RAF kinase; MEK: mitogen-activated protein kinase; MAPK: mitogen-activated protein kinase; CREB: cAMP response element-binding protein; MYC: c-MYC; HIF: hypoxia-inducible factors; NF-κB: nuclear factor kappa B.

ROS molecules are generally generated from incompletely reduced oxygen, which accepts electrons from the ETCs of the mitochondria [190]. During the ETC process, some electrons escape and settle to O_2_, which become ROS. ROS generated by the leakage from the ETC is the most substantial source of cellular ROS (Figure 4). However, in addition to the ETC-generated ROS, the cytochrome P450 enzymes (CYPs) and NADPH oxidases (NOXs) also produce them. CYPs are a superfamily of enzymes that contain hemes as a cofactor and that function as monooxygenases, exhibiting a broad range of substrate specificities [191]. CYPs accidentally produce ROS as byproducts during their oxidation reactions, as in the case of the ETC [192]. Unlike the ROS from the ETC and CYPs, NOXs produce ROS as their intended enzymatic product rather than as a byproduct [193]. NOXs are six-transmembrane proteins with a conserved core element that contains four heme-binding histidines to transfer electrons to oxygen for the production of ROS [193]. Researchers have identified six members of the NOX family, including NOX1, NOX3, NOX4, NOX5, DUOX1, and DUOX2 [194]. These enzymes are expressed in a cell-specific manner and constitute one of the major sources of the intracellular ROS beside the mitochondria. Researchers initially identified the function of NOXs (NOX1) in the membrane of phagocytic cells, where the ROS production led to the destruction of pathogens by immune cells [195,196]. Later, researchers elucidated that NOXs play various functions in post-translational modifications, lipids, the calcium level, the oxygen sensor, and metabolic reprograming, as well as cell differentiation, transformation, growth, and death in normal cells depending on the cell type [197,198,199]. As described above for glucose and lipid metabolism, the oxidative pathways of G6PDH, 6PGLDH, ME1, and IDH1, which produce NADPH, are elevated. The increased concentration of NADPH drives the enzymatic activities of NOXs to increase the production of ROS. In this context, NOX-derived ROS are a logical contributor to the elevated concentrations of ROS in cancer cells. In fact, cancer cells ubiquitously up-regulate the expression of NOX4, and the up-regulated NOX4 enhances the production of ROS [200].

The emergence of cancers starts with the mutagenic conversion of proto-oncogenes to oncogenes. The transformation of proto-oncogenes such as RAS, RAF, MEK, ERK, and AKT to oncogenes due to chemical, physical, or genetic mutations stimulates the cell proliferation in early cancer by activating the signaling pathways of the unbridled cell proliferation, and by inactivating the signaling pathways of the apoptosis suppression [201]. Cell proliferation demands that ATP is mostly generated from mitochondrial oxidative phosphorylation; thus, the oxidative phosphorylation pathway is upregulated in fast-dividing cells. Because of electron leakage, which is the source of ROS is an inevitable consequence of the OXPHOS pathway, the upregulation of mitochondrial OXPHOS in early cancer cells leads to an increased production of ROS [202,203]. The structure of cancer tissue also contributes to the increased production of ROS. The uncontrolled, rapid growth of cancer cells leads to the formation of unorganized cell masses, which make it impossible to develop functional neovascularization, as in the case of normal tissues. The lack of a proper vascular system in cancer gives rise to an inadequate blood supply, which results in chronic hypoxia [204]. The generation of ROS, as well as the NF-κB-dependent transcription, are facilitated in the hypoxic condition [205,206]. Moreover, the hypoxia-sensitive transcription factor HIF-1α activates the expression of NOX4 [207]. Conversely, H_2_O_2_ generated from ETS, CYPs, and NOX4 stabilizes HIF-1α for an even higher increase in the production of ROS [208,209,210]. The chain reaction between H_2_O_2_ and NOX4 that is initiated by hypoxia seems to make NOX4 a key player in ROS generation in cancer.

Both the increased OXPHOS pathway and the development of the chronic hypoxic condition inevitably contribute to the increased production of ROS in cancer. The increased ROS stimulate the proximal activation of the PI3K/Akt and MAPK/ERK signaling pathways, which stimulate the early cancer cells to more rapidly proliferate [211,212,213,214,215]. In addition to the activation of the signaling pathways, the increased ROS activate the HIF and NF-kB transcription factors [215]. These transcription factors also contribute to the stimulation of cell proliferation. Finally, the increased ROS apodictically mutate genomic DNA through their oxidative damaging capability, which contributes to genome instability [216]. Overall, ROS play the key role in the evolution of early cancer cells into more malignant cancerous cells as they progress into the later stage, while oncogenes drive the transformation of cancer cells from normal cells.

The high redox state endows cancer cells with another unique property. Most redox enzymes contain hemes with a central iron ion at their protoporphyrin cores as a cofactor [217]. Because of the high demand for redox enzymes, one of the key characteristics of cancer cells is that they actively absorb hemin, protoporphyrin, and iron [218,219,220]. Cancer cells increase the cellular concentrations of hemin and protoporphyrin by both the activate synthesis of the compounds as a result of the activated RAS/MEK pathway [221], and the upregulation of two ABC transporters: ABCG2 and ABCB6 [222,223]. Porphyrin compounds, such as hemin, selectively accumulate in cancer cells in vivo [224]. Because of the trophic feature of porphyrin compounds to cancer cells, researchers are actively developing a technology that uses a porphyrin compound as a targeting moiety in anticancer drug delivery.

## 6. Perspective on Future of Anti-Cancer Drug Development and Conclusions

The metabolism of cancer cells and their subsequent signaling transductions are altered in comparison with normal cells to support the elevated energy demand, as well as the acquisition and maintenance of their malignant properties. The metabolic alterations, such as the Warburg effect, ROS increase, and glutamine dependence, are the prominent features after the transformation of normal cells into cancer cells. Because researchers discovered that the Warburg effect and ROS increase first, there have been countless attempts to develop anti-cancer drugs that target them (Table 1). However, these attempts have fallen short of expectations.

Despite the fact that glutamine dependence is a hallmark of cancer cell metabolism, the significance of glutamine dependance in cancer metabolism has been realized relatively recently compared to the Warburg effect and ROS increase. As a result, the drug development of anti-cancer drugs targeting the glutamine dependence has recently started. CB-839, Sirpiglenastat (DRP-104) and DON showed excellent therapeutic efficacies in the clinical trials [285,291,294,295]. The clinical successes promoted a burst of development of anticancer drugs targeting glutamine dependence by inhibiting glutaminase or the glutamine transporter (Table 1). Unlike the anticancer drugs targeting the Warburg effect and ROS increase, these drugs do not affect the metabolism of normal cells because glutamine dependence is the uniquely limited to cancer cells. Because normal cells are not addicted to glutamine, the anticancer drugs targeting glutamine dependence showed a negligible side effect in clinical trial, which means that the drugs are ideal for combination therapies with other anticancer drugs. Because there is a plethora of cytotoxic and immunologic anticancer drugs, the development of combination therapies that use anticancer drugs that target glutamine inhibition with cytotoxic or immunologic anticancer drugs should be actively investigated as potential novel cancer treatment strategies.

## Figures and Tables

**Table 1:** Therapeutic strategies for targeting cancer metabolism.

Agent	Pathway/Target	Mode of Action	Stage of Drug Development	Reference
Targeting Aerobic Glycolysis and Pyruvate Metabolism				
WZB117	GLUT1	Inhibition of glucose uptake	Preclinical	[225]
STF-31	GLUT1		Preclinical	[226]
Fasentin	GLUT1		Preclinical	[227,228]
Polyphenol phloretin	GLUT2		Preclinical	[229,230]
Flavonoids (myricetin, fisetine, quercetin, isogquercitrin)	GLUT2		Preclinical or in market for the treatment of hematopoietic/lymphoid or solid cancer	[231]
Ritonavir	GLUT4		On the market for the treatment of multiple myeloma	[232,233]
KU-55933	ATM/GLUT1		Preclinical	[234]
Resveratrol	Akt/mTOR/GLUT1		Preclinical	[235]
2-deoxyglucose (2-DG)	Hexokinase 2 (HK2)	Inhibition of glycolysis	Clinical trial phase 2 for the treatment of advanced cancer and hormone refractory prostate cancer	[236,237]
3-bromopyruvate (BrPA)			Preclinical	[238,239]
Lonidamine			Clinical trial phase 3 for the treatment of breast cancer	[240,241]
Genistein (GEN)-27			On the market for the treatment of breast cancer	[242,243]
Benserazide			On the market for the treatment of colon cancer	[244]
Natural products (astragalin, resveratrol, chrysin)			Preclinical or in market	[245,246]
3PO	6-phosphofructo-2-kinase (PFKB3)	Inhibition of glycolysis	Preclinical	[247,248]
PFK15			Preclinical	[248]
Koningic acid (KA)	Glyceraldehyde-3-phosphate dehydrogenase (GAPDH)	Inhibition of glycolysis	Preclinical	[249,250]
DC-5163			Preclinical	[251]
Methylglyoxal (MG)			Clinical trial phase 3 for the treatment of myelocytic leukemia and lymphomatous disease	[252]
TT-232	Pyruvate kinase type M2 (PKM2)	Inhibition of glycolysis and induction of apoptosis	Clinical trial phase 2 for the treatment of renal cell carcinoma	[253]
Shikonin			Preclinical	[254]
Vitamin K(3) and K(5)			Clinical trial phase 1	[255]
Oleanolic acid (OA)			Preclinical	[256]
CPI-613	Pyruvate dehydrogenase (PDH) and α-ketoglutarate dehydrogenase	Inhibition of mitochondrial metabolism	Clinical trial phase 3 for the treatment of pancreatic cancer	[257,258]
Dichloroacetate (DCA)	Pyruvate dehydrogenase kinase (PDK)	Shifting from glycolysis to oxidative phosphorylation	On the market for the treatment of glioblastoma multiforme	[259,260,261]
Mitaplatin			Preclinical	[262,263]
Betulinic acid (BA)			Clinical trial phase 2 for the treatment of colon cancer	[264]
Oxamate	Lactate dehydrogenase A (LDHA)	Pyruvate competitive	Preclinical	[265,266]
Gossypol		NADH competitive	Phase 1~3 for the treatment of glioblastoma multiforme	[267,268]
FX11		NADH competitive	Preclinical	[269]
N-hydroxyindoles (NHI)		Pyruvate and NADH competitive	Preclinical	[270]
Galloflavin (GF)		Binding of free enzyme	Preclinical	[271]
AZD3965	Monocarboxylate transporter (MCT1)	Inhibition of glycolysis	Clinical trial phase 1	[272]
Targeting Pentose Phosphate Pathway				
Dehydroepiandrosterone (DHEA)	Glucose-6-phosphate dehydrogenase (G6PD)	Inhibition of glycolysis and induction of apoptosis	On the market for the treatment of breast cancer and prostate cancer	[273,274]
6-aminonicotinamide (6-AN)			Preclinical	[275]
Polydatin			Clinical trial phase 2 for the treatment of hepatocellular carcinoma	[276,277]
Targeting Serine Biosynthesis Pathway				
CBR-5884	3-phosphoglycerate dehydrogenase (PHGDH)	Inhibition of de novo serine synthesis	Preclinical	[278]
NCT-503			Preclinical	[279]
Targeting Glutamine Metabolism and Mitochondrial TCA cycle				
V-9302	ASCT2 (SCL1A5)	Inhibition of glutamine transport	Preclinical	[280,281]
GPNA			Preclinical	[282]
Benzylcysteine			Preclinical	[283]
Benzylserine			Preclinical	[284]
BCH	LAT1 (SCL7A5)	Inhibition of glutamine-essential amino acid transport	Preclinical	[285]
DON	Glutamine-utilizing enzymes, including glutaminase	Glutamine antagonist	Clinical trial phase 3 for the treatment of advanced refractory solid tumors	[286]
JHU-083			Preclinical	[287,288]
Rais-5C			Preclinical	[289]
Nedelcovych-13d			Preclinical	[290]
JHU-395			Preclinical	[291]
Sirpiglenastat (DRP-104)			Clinical trial phase 2 for the treatment of glioblastoma	[292]
BPTES	Glutaminase (GLS1)	Allosteric inhibitor mTOR signaling downregulation and inhibition of TCA anaplerosis	Preclinical	[293,294]
CB-839			Clinical trial phase 2 for the treatment of triple-negative breast cancer	[295,296]
Compound 968			Preclinical	[297,298]
Alkyl benzoquinones	Glutaminase (GLS2)	mTOR signaling downregulation and active autophagy	Preclinical	[299]
Thiazolidine-2,4-dione	Glutaminase (GLS1 and GLS2)	Moderately more selective for GLS1 than GLS2	On the market for the treatment of lung cancer	[300]
Amino oxyacetate (AOA)	Transaminase	Glutaminolysis inhibition in Myc driven tumors	Preclinical	[131]
Epigallocatechin gallate (EGCG)	Glutamate dehydrogenase (GLUD1)	Decreased glutathione peroxidase activity	On the market for the treatment of glioblastoma	[131,301]
R162			Preclinical	[302]
UK5099	Mitochondrial pyruvate carrier (MPC)	Inhibition of pyruvate transport	Preclinical	[303]
Targeting Fatty acid Metabolism				
Etomoxir	Carnitine palmitoyl transferase 1 (CPT1)	Inhibition of fatty acid oxidation	Preclinical	[304,305,306]
BMS-303142	ATP citrate lyase (ACLY)	Inhibition of de novo lipid synthesis	Preclinical	[307]
SB-204990			Preclinical	[308]
Soraphen A	Acetyl-CoA carboxylase (ACC)		Preclinical	[309]
ND-646			Preclinical	[310]
Cerulenin	Fatty acid synthase (FASN)		Preclinical	[311,312]
TVB-2640			Clinical trial phase 2 for the treatment of lung cancer, breast cancer, ovarian cancer	[313]
Orlistat			On the market for the treatment of gliomas and T cell lymphoma	[314,315]
C75			Clinical trial phase 2 for the treatment of prostate cancer	[316]
CAY10566	Stearoyl-CoA desaturase 1 (SCD1)		Preclinical	[317]
A939572			Preclinical	[318,319]
Fatostatin	Sterol regulatory element binding protein 1 (SREBP-1)		Preclinical	[320,321]
Betulin			Preclinical	[322]
PF-429242			Preclinical	[323]
MSI-1			Preclinical	[324]
Targeting Isocitrate Dehydrogenase (IDH)				
AG-120 (ivosidenib)	IDH1	DNA and histone hypermethylation	Clinical trial phase 3 for the treatment of cholangiocarcinoma	[325,326]
AG-221 (enasidenib)	IDH2		Clinical trial phase 3 for the treatment of refractory and relapsed acute myeloid leukemia	[327,328,329]
IDH305	IDH1		Clinical trial phase 2 for the treatment of leukemia	[330]
AG-881 (vorasidenib)	IDH1/2		Clinical trial phase 3 for the treatment of glioma	[331,332]
FT-2102 (olutasidenib)	IDH1		Clinical trial phase 2 for the treatment of relapsed or refractory glioma	[333,334]
BAY1436032	IDH1		Clinical trial phase 1	[335]

## Data Availability

Not applicable.
